# Multidimensional poverty measure and analysis: a case study from Hechi City, China

**DOI:** 10.1186/s40064-016-2192-7

**Published:** 2016-05-17

**Authors:** Yanhui Wang, Baixue Wang

**Affiliations:** 3D Information Collection and Application Key Lab of Education Ministry, College of Resource Environment and Tourism, Capital Normal University, No. 105 West Third Ring North Road, Haidian District, Beijing, 100048 China; Department of Geography and Anthropology, Louisiana State University, Baton Rouge, LA USA

**Keywords:** Multidimensional poverty measurement, Poverty identification indicators, Spatial pattern, Diversity analysis, Geographical constraint, Hechi City

## Abstract

Aiming at the anti-poverty outline of China and the human–environment sustainable development, we propose a multidimensional poverty measure and analysis methodology for measuring the poverty-stricken counties and their contributing factors. We build a set of multidimensional poverty indicators with Chinese characteristics, integrating A–F double cutoffs, dimensional aggregation and decomposition approach, and GIS spatial analysis to evaluate the poor’s multidimensional poverty characteristics under different geographic and socioeconomic conditions. The case study from 11 counties of Hechi City shows that, firstly, each county existed at least four respects of poverty, and overall the poverty level showed the spatial pattern of surrounding higher versus middle lower. Secondly, three main poverty contributing factors were unsafe housing, family health and adults’ illiteracy, while the secondary factors include fuel type and children enrollment rate, etc., generally demonstrating strong autocorrelation; in terms of poverty degree, the western of the research area shows a significant aggregation effect, whereas the central and the eastern represent significant spatial heterogeneous distribution. Thirdly, under three kinds of socioeconomic classifications, the intra-classification diversities of *H*, *A*, and *MPI* are greater than their inter-classification ones, while each of the three indexes has a positive correlation with both the rocky desertification degree and topographic fragmentation degree, respectively. This study could help policymakers better understand the local poverty by identifying the poor, locating them and describing their characteristics, so as to take differentiated poverty alleviation measures according to specific conditions of each county.

## Background

 As the world’s most populous developing country, China had experienced various poverty alleviation and development programs in rural areas, resulting in that the absolute poverty population had dropped from 94.22 million in 2000 to 26.88 million in 2010, and the poverty rate had fell from 10.2 to 2.8 %, according to National Statistics Bureau of China (2012). However, due to the complex geographic and socioeconomic conditions of rural China, the development gaps among different regions have been increasing, and the region-related characteristic have also been obvious (Wang and Qian 2015), indicating that China’s previous income-based poverty identification policy had to overcome great difficulties to precisely target those poverty-stricken households (Lu 2012). Therefore, the policymakers need specific information and tools to systematically identify those areas where development lags and where the poor live (Henninger and Snel 2002; Okwi et al. 2007), and further specifically target them to solve the poverty one by one with special policies.

In this context, China have been adjusting its poverty elimination policy from a purely monetary perspective to a more multidimensional view on poverty (Wang and Alkire 2009). It announced the ‘China’s rural poverty alleviation and development Outline (2011–2020)’ (hereinafter referred to as the ‘New Outline’) in 2011, assigning 14 contiguous destitute areas to act as the main battlefields to basically eliminate absolute poverty in 2020. In this policy document, Chinese government proposed the specified anti-poverty goal of the poor households that the poor themselves need not have to worry about their eating and wearing in the future; in addition, their obligatory education, basic medical treatment, and basic housing should be guaranteed by the government. In 2014, when the precisely targeting and identifying of the poor in rural areas is still a primary problem to be solved in the new anti-poverty stage, China further released a new policy of ‘Precise Poverty Reduction’ aiming at the implement of ‘New Outline’, and emphasized the precondition of accurately targeting the poor and their poverty factors to achieve the objective of building a full well-off society. However, driven by Chinese historical routine of national economic-core poverty identification, the previous practice on identifying the poor had still been done based on a single standard of economic income, which obviously ignored the basic rights of the poor in terms of housing, health, education, etc. In addition, the authenticity and reliability of income data is limited, resulting in that the traditional income-based method has been unable to meet China’s strategic needs of currently precise poverty alleviation. Therefore, new methods are needed to be introduced to identify the complex and multidimensional poverty in rural China, while multidimensional measures provide such an alternative lens through which poverty may be viewed and understood, as it is quite a departure for traditional unidimensional and multidimensional poverty measurement-particularly with respect to identification step-further elaboration may be warranted (Alkire and Foster 2011).

In this context, from the views of multidimensional poverty, bringing housing, health, education and other indicators into the evaluation system to comprehensively measure and analyze the rural poverty has increasingly been becoming a hot topic of domestic and foreign research (Wang and Alkire 2009; Alkire and Foster 2011; Guedes et al. 2012; Wang et al. 2013). Accordingly, in accordance with China’s current precise anti-poverty policy, the objective of this study is to develop a multidimensional poverty measure and analysis methodology, taking typical area of rural China as one case to accurately measure multidimensional poverty and its contributing factors under given socioeconomic and geographical conditions.

## Related work

Internationally, the previous poverty identification was often designed from the unidimensional view, e.g., according to the most popular poverty standard line of $ 1.25 per person per day developed by World Bank, or the poverty line of 2300 Chinese RMB yuan per person per year in China in 2011; however, this method had showed its distinct limitation due to its immoderate simplicity (Wang and Qian 2015).

With the increasing understanding that poverty is of multidimensional and dynamic natures, many studies had responded with new measures and tools that comprehensively measure poverty to the strong demands of governments and international communities (Anand and Sen 1997; Bourguignon and Chakravarty 2003; Maasoumi and Lugo 2008; Alkire and Foster 2011; Guedes et al. 2012). For examples, poverty was measured by use of Human Development Index (UNDP 2000), and also by Multidimensional Poverty Index (*MPI*) proposed by Alkire and Foster (2010), both of them stressing that human poverty was caused by inequalities of their achieved rights and abilities, such as education, health, job, policy and so on. This view has increasingly been becoming a hot topic of domestic and foreign research, and special practice had also been done aiming at different study areas in different countries and regions (Anand and Sen 1997; Bourguignon and Chakravarty 2003; Thomas et al. 2009; Alkire and Foster 2011; Gilvan et al. 2012).

On the other hand, since adoption of a multidimensional approach to deprivation poses the challenge of understanding the interaction between different dimensions (Atkinson 2003), the need for such a multidimensional approach to the robustness measurement of multidimensional inequality had also been emphasized in other literatures (Atkinson and Bourguignon 1982; Tsui 1985; Maasoumi 1986; Sen 1999; Bourguignon and Chakravarty 2003). Aiming at the ‘right’ poverty-line that should be concerned with the union of all those deprived on at least one dimension or with the intersection of those deprived on all dimensions, diverse approaches to the study of poverty appear to fall into two categories: non-axiomatic approach in which different indicators are combined in order to obtain a multidimensional index (Betti et al. 2015), and axiomatic approaches that had been developed by Chakravarty et al. (1998), Bourguignon and Chakravarty (2003). Atkinson (2003) brought out key features of different approaches and sets them in a common framework. Duclos et al. (2001) compared union and intersection method by which to decide who is poor in multiple dimensions, demonstrating how to check whether the comparisons are robust to aggregation procedures and to the choice of multidimensional poverty lines. Additionally, the ‘counting’ approach, widely used in applied studies (UNDP 1997; Wang et al. 2013), is devoted to counting the number of dimensions in which people suffer deprivation, in which both union and intersection conditions may be necessary. Considering poverty as a matter of degree rather than an attribute that is simply present or absent for individuals in the population, Cheli and Lemmi (1995), and Betti et al. (2015) adopted a fuzzy set approach to draw a distinction between those who adopt a *union* approach and those who use an *intersection* measure.

On the other hand, according to some authors (UNDP 1997; Wang et al. 2013; Wang et al. 2015), these weights of the indicators should be equal while the composite welfare index is an average of the responses to the different variables. On the other hand, some others suggested that the weights allocated to the indicators must vary as a function of their contribution to welfare, and they developed such methodologies to response to this view, e.g., entropy approach (Maasoumi 1999), multiple correspondence analysis (Ningaye and Njong 2015), fuzzy set approach (Cheli and Lemmi 1995; Njong and Baye 2010; Betti et al. 2015), and so on.

Alkire and Foster (2011) attempted to offer a practical A–F approach as the methodology of measurement and analysis multidimensional poverty, identifying people as poor depending upon achievements of household members. A–F measure with desirable axiomatic properties could reflect the breadth, intensity and components of deprivations and improve the counting-based headcount measures of multidimensional poverty, presenting indicators of multiple dimensions with a single summary index that can be broken down among the dimensions and different groups (Alkire and Roche 2011; Wang et al. 2015). It has also been widely applied to the investigations of the multidimensional poverty status in India, China and Latin American countries (Alkire and Seth 2013; Yu 2013; Battiston et al. 2013). In regards to the weight of these dimensions, most of the indexes apply equal weights implicitly or explicitly (Alkire and Roche 2011; Wang et al. 2013; Qi and Wu 2015; Wang et al. 2015). To simplify interpretation, Alkire and Roche (2011) argued that equal weight was plausible and commonly adopted.

Meanwhile, following the international multidimensional poverty view, some studies had also been done to aim at China’s multidimensional poverty identification by using A–F method, i.e., Wang and Alkire (2009) collected sampling data from China Health and Nutrition Survey of 2006, using the eight indicators of health, education, housing, and living standard to carry out the study area’s multidimensional poverty estimations. Li (2009) implemented the four dimensions of education, health, environment and consumption to perform a poverty measurement for 30 poor counties. Wang et al. (2013) conducted the village-level case study of multidimensional poverty. Multidimensional child poverty index and its dynamic changes in China were also studied by Qi and Wu (2015), and Wang et al. (2015). Maasoumi and Xu (2015) combined multidimensional welfare analysis and entropy metrics to derive not only the best relative weights but also substitution degree among different attributes to construct multidimensional indices of well-being with CHIPS 2002 data. Recently, Yang and Mukhopadhaya (2016) measured multidimensional poverty in China at the national, rural–urban, regional and provincial levels using the China Family Panel Studies data from 2010, and observed that when they adopted four kinds of different methods to measure multidimensional poverty, the variation of weights did not change the results much.

Although the above multidimensional poverty research had made great strides, China’s poverty evaluation indicator system has not completed yet due to the absence of an objective standard. Since scholars often pursued the quantity and all-sided indexes during measuring poverty, however, most of them have not delve into the detailed poverty types and poverty contributing factors, resulting in inconsistence with the goal of China’ existing precisely targeting the poor. What’ more, most of the cases’ data source had their natural limitations, the reason is that the sampling principle commonly used for poor households’ information collection was 120 households per county, however, this would overlook the differences among various poor households, insufficiently depicting various characteristics of China’s poverty, due to the higher population density, huger gaps among rural areas, complex contributing poverty factors, and special anti-poverty policy. As a result of the less sample data, it often caused deviations between the measurement results and the actual poverty (Li et al. 2005; Hayati et al. 2006; Islam and Maitra 2012; Thongdara et al. 2012).

On the other hand, Poverty is also geographical related, geographical environment has a significant influence on the state of poverty particularly in mountainous regions (Madulu 2005; Vista and Murayama 2011). So considering the impact of the natural environment on poverty, the geographical distribution of the poor has also become another current hot topic. For examples, some studies explored the case area’s poverty determinants in terms of poverty fragility (Christiaensen and Subbarao 2005; Islam and Maitra 2012). Some attempted to use GIS to evaluate poverty (Hentschel et al. 2000; Akinyemi 2008; Wang et al. 2013). However, most research of the above focused on the perspective of sociology and economics, seldom quantitatively considering the influence of geographical environment on poverty under different geographic constraints.

Therefore, to address the above problems to fulfill China’s precise poverty reduction strategy, taking the whole poor households’ census data from Hechi City, China, as our test case, we combines quantitative GIS analysis with RS digital image processing technology, attempting to develop a multidimensional poverty measurement and analysis methodology to assess each county’s poverty level and the poverty contributing factors. According to study area’s geographic and socioeconomic characteristics, the poverty analysis could help policymakers better understand the local poverty by identifying the poor, locating them and describing their characteristics, so as to take differentiated poverty alleviation measures according to specific conditions of each county.

## Research area and data sources

### Research area

Hechi City belongs to Guangxi Zhuang Autonomous Region, China, and is a part of Yunnan and Guangxi and Guizhou rocky desertification areas amongst the 14 contiguous destitute areas. As a historic revolutionary base, gathered minority, border and mountainous region, Hechi is underdeveloped with the typical Chinese poverty-stricken characteristics. In other words, its conflict among population, resources and environment is very prominent, e.g., it has a total population of 4,500,000, mainly made up of Zhuang, and the minority nationalities account for 83.67 % of the total population. It is mountainous, mainly distributed at the border, with the diverse terrain that is high in the northeast and low in the southeast. On the other hand, with its widespread karst geomorphology, it is considered as the worst rocky desertification and fragmentation area in China, the area of rocky desertification being 722,600 hectares.

**Fig. 1:**
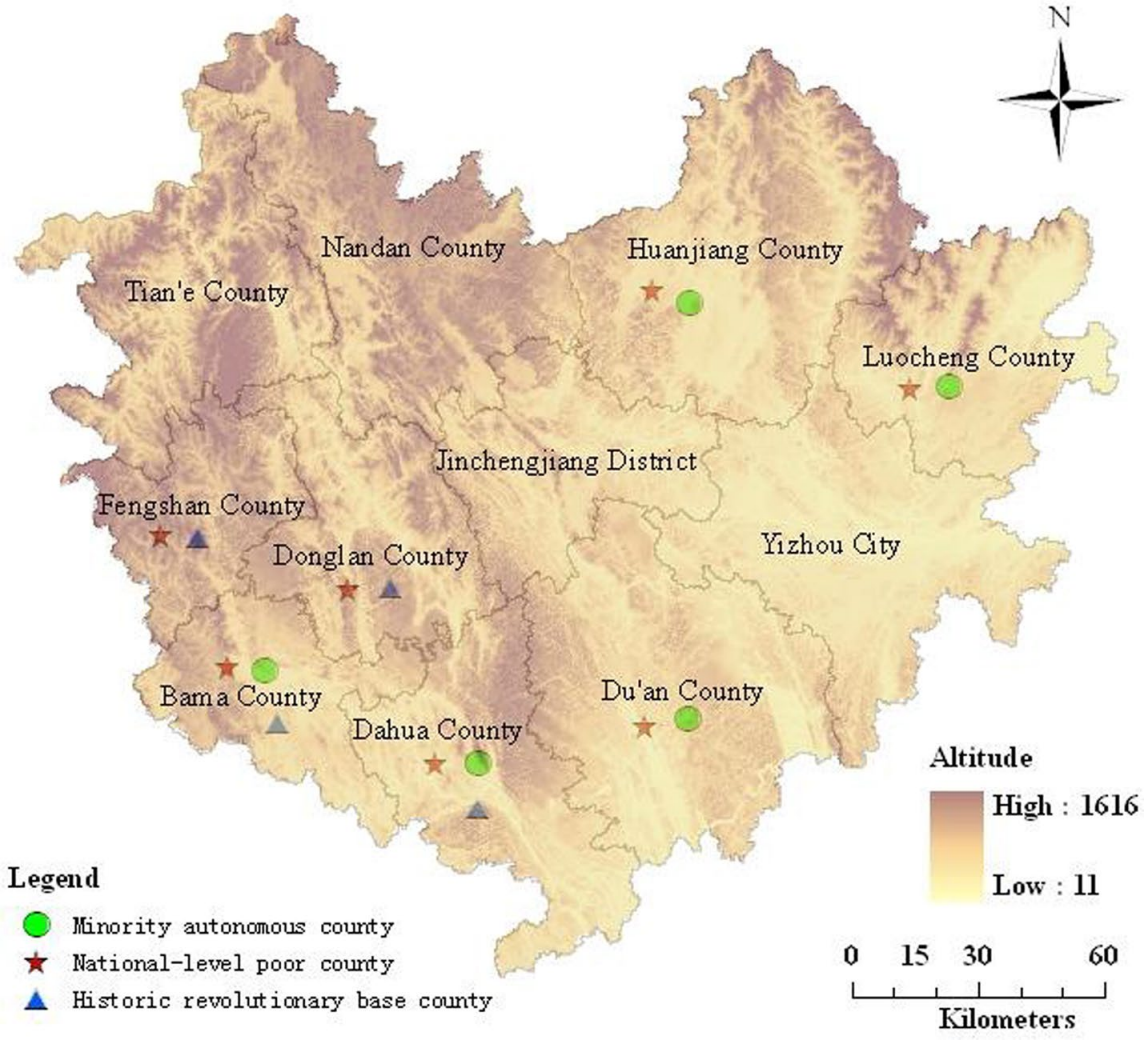
Illustration of the study area

Further specifically, 11 counties are under the jurisdiction of Hechi City, e.g., Jincheng, Yizhou, Luocheng, and Bama, etc. Among which, there are 7 ones belonging to the national key poverty-stricken counties, and the other 4 counties are classified as province-level ones. On the other hand, there are 5 minority autonomous counties and 4 historic revolutionary base ones. As shown in Fig. 1, ‘national-level’ refers to that the county belongs to national-level poverty-stricken counties; ‘revolutionary base’ refers to that the county belongs to historic revolutionary bases; ‘autonomous county’ refers to minority autonomous county; ‘other’ refers to counties that do not belong to any kind of above.

### Data sources and preprocessing

The data collected for this study includes socioeconomic dataset and basic geographic information dataset. The former stems from 2013 census archiving data of rural poor households, i.e., household sheets, provided by official poverty alleviation department of Hechi City, covering a wide range of headcount information about each households, e.g., housing conditions, production conditions and living conditions, and all adding up to 1578 villages and approximately 1,100,000 individuals. The latter geographic dataset includes mainly the 1:250,000 geographic data, national 90 m DEM data, and landsat8 raster image of the same period in the study area.

These data are pre-processed before putting into use, e.g., by eliminating unreasonable socioeconomic data, spatial data’s georeferencing, clipping, as well as joining spatial and corresponding socioeconomic data. In addition, as a result of interactions between vulnerable ecological environment and unreasonable human activities, the rocky desertification degrees of the study area are calculated from landsat8 image by using supervised classification and utilizing ENVI software to combine 4, 3, 2 wave bands for pseudocolor synthesis.

## Research methods

In light of the complex poverty characteristics of the study area, this article constructs a set of multidimensional poverty measurement indicators and evaluation methodology with Chinese characteristics, taking the rural poverty-stricken households as the evaluation unit, and county as the output unit, designing multidimensional measure indicators, adopting A–F identification and spatial geographic statistics to compare and analyze the diversities of poverty characteristics, as well as their spatial distribution under different natural and socioeconomic situations.

### Multidimensional poverty measurement

According to Alkire and Foster (2011), A–F ‘dual cutoff’ identification approach can be seen as a general framework for measuring multidimensional poverty since many key decisions are left to us. These includes the selection of dimensions and indicators, derivation cutoffs (to determine when a person is deprived in a dimension or indicator), weights (to indicate the relative importance of the different deprivations), and a poverty cutoff (to determine when a person has enough deprivations to be considered to be poor). So, our work in this section is to give the response to the above to fit the purpose of the precise poverty measure and intervenes, and as well as to embody normative judgements regarding what it means to be poor in the study area.

#### Multidimensional poverty measure indicator system

The principles by which we build multidimensional poverty measure indicator system are as follows, (1) responding to the implement demand of Chinese precise poverty alleviation of the ‘New Outline’; (2) In accordance with the indeed requirements of the scientific nature, typicality, data availability, policy relevance and practicality for building common indicators; (3) Referring to the poverty-related identification methodology put forward by a wide range of research scholars (Li 2009; Ravallion 2011; Lu 2012; Hu and Ou 2013).

To be specified, after the collection of the indicator candidates from the census archiving data of households, we integrates the above principles with Chinese special poverty reduction objective of ensuring that the poor farmers do not have to worry about food and clothing and they have to be guaranteed for compulsory education, basic medical treatment and housing, and filter out four dimensions, namely, housing, health, education, living conditions. Then, taken income as the independent variable, logistic regression is used here to check the validity of each deprivation dimension by investigating whether or not dimensional deprivation are significantly associated with income that are known to be correlated with poverty. Results showing all the coefficients are statistically significant which guarantee the validity of all the four dimensions. Besides, to objectively eliminate redundant and non-critical ones, each candidate in the above four dimensions goes through the threshold sensitivity examination of the correlation coefficient by use of the exploratory factor analysis method in SPSS software environment. At last, a set of multidimensional poverty measure indicators for Hechi City is constructed, as shown in Table 1, there are altogether 4 dimensions and 10 basic indicators in the system. Where, as the basis for determining who is deprived and in which indicator, the derivation cutoff depends on anti-poverty development goal under Chinese current special conditions, and its special definition is given in the Table to satisfy dimensional monotonicity and key properties for policy and analysis, e.g., one farmer is poor if his health is weakly below the cutoff.

**Table 1:** Measurement indices of multidimensional poverty

Dimension	Indicator	Deprivation cutoff	Weight
Housing (1/4)	House safety	Given brick and concrete structure is not dangerous, the assignment is 0, otherwise 1	1/4
Health (1/4)	Members’ health	In one household, if there is at least one member under a serious illness, the assignment is 1, otherwise 0	1/4
Education (1/4)	Adults’ illiteracy	In one household, if there is at least one illiterate adult, the assignment is 1, otherwise 0	1/8
	School-age children’ enrollment	In one household, if there is a 6–16 aged child out of school, the assignment is 1, otherwise 0	1/8
Living conditions (1/4)	Drinking water’ safety	Given the water from shallow well, deep well, or tap water is safe, assignment 0, otherwise 1	1/24
	Drinking water’ availability	If one household can’t get sufficient drinking water in a convenient way, the assignment is 1, otherwise 0	1/24
	Sanitary facilities	If one household have a water toilet, the assignment is 0, otherwise 1	1/24
	Electricity access	If one household can use electricity, the assignment is 0, otherwise 1	1/24
	Broadcasting access	If one household can use the broadcasting, the assignment is 0, otherwise 1	1/24
	Fuel type	If one household can only use dirty energy fuel, e.g., firewood, straw, etc., the assignment is 1, otherwise 0	1/24

In regards to the weight of these dimensions in A–F method, considering that the policy-related criterion that each aspect of households’ living conditions should be synchronously improved to keep the same pace with building national moderately prosperous society of China, as well as that the equal weighting method is also a commonly adopted scheme in international MPI (Wang et al. 2015), this paper assigned equal weight to all four dimensions as well as the indicators within each dimension. To be specified, each dimension has the same weight value of 1/4, and each indicator within each dimension shares the same weights of the dimension.

#### Multidimensional poverty measure methods

A–F method describes how poor people are identified by using ‘dual cutoffs’ (Alkire and Foster 2011), so this article introduces the A–F dual cutoffs poverty line to identify that a person is poor if multiply deprived enough. Here, dual cutoffs means deprivation cutoff and poverty cutoff between the target group and the remaining population, respectively indicating who is deprived and in which indicator, and whether a person is deprived enough to be called poor. To be specified, this article conducts multidimensional poverty identification for the targeting of ‘who is poor?’, addressing whether each indicator of one household has been deprived by employing the deprivation cutoff, as well as whether the household is overall multidimensional poverty-stricken with the help of poverty cutoff.

Further, during the dimensional poverty measurement, A–F dimensional aggregation and decomposition methodology is also adopted to respectively reflect the overall level of poverty in a given poverty line, i.e., addressing ‘how much poverty there is overall?’ for evaluation and monitoring, the joint distribution of deprivations, as well as the breakdown by dimension after identification. As a result, households’ deprived capabilities are identified, in which the multidimensional poverty incidence (*H*), average deprivation quota (*A*), multidimensional poverty index (*MPI*), and the contribution degree of each indicator (*C*) are measured.

**Fig. 2:**
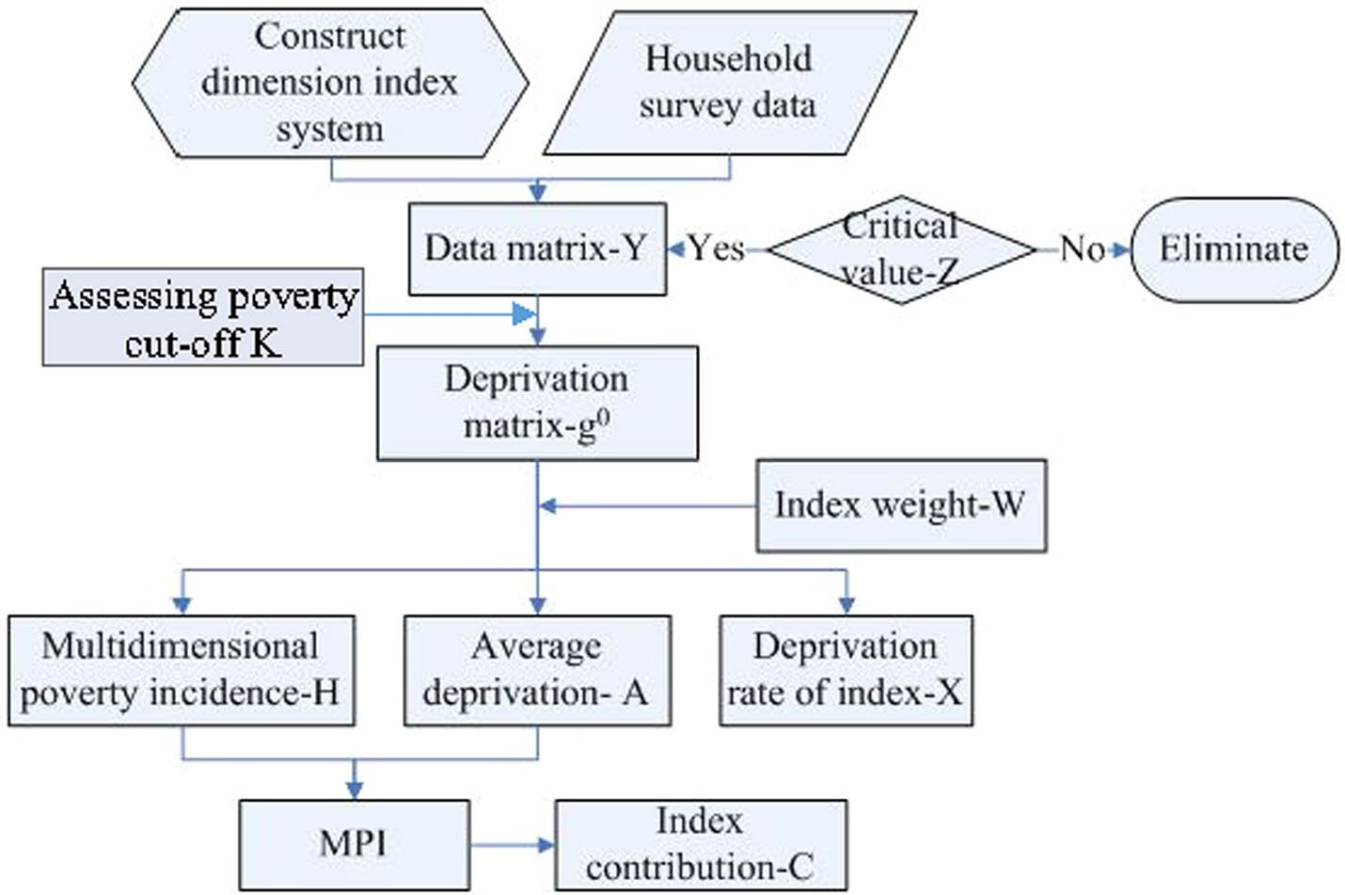
Poverty measurement flow

So this article takes county as the output unit to conduct poverty analysis, synthesizing the multidimensional measure results of households in each county. The whole flows are shown in Fig. 2, as well as the specific explanation in Table 2.

**Table 2:** Interpretation of specific variables

Variable name	Interpretation
Recall achievement data matrix-*Y*	*Y (n* × *d)*, used to store the indicator information of households, *n* represents the number of the individuals, and *d* represents the number of the indicators
Censored deprivation matrix-*g* ^0^	*g* ^0^ *(n* × *d)*, used to store the identified situation of poor households being deprived. If one household is deprived in the given indicator, assigning 1, otherwise, 0
Deprivation cutoff-*Z*	By which to determine whether one household is poor or not from the view of a certain indicator
Poverty cutoff-*K*	By which to determine whether the households are in multidimensional poverty or not, i.e., if the number of indicators that one household is deprived is greater than that *K*, then it can be considered that the household is under *K*- multidimensional poverty
Multidimensional headcount ratio-*H*	The ratio of multidimensional poverty population to the total population, seeing the formula: *H* = *q/n*, where, *q* represents the multidimensional poverty population; *n* indicates the total population of the region
Average deprivation share among the poor-*A*	The average number of multidimensional poverty population, also called Intensity of multidimensional poverty deprivation, seeing the formula: $A = \frac{{\sum\nolimits_{i = 1}^{n} {c_{i} (k)} }}{q}$ where, *c*_*i*_ *(k)* indicates that the number of indicators that individual *i* is deprived in the case of poverty threshold *k*; *q* denotes the multidimensional poverty population
Multidimensional Poverty Index-*MPI*	The comprehensive index of the poverty degree in the given region, obtained by the formula: *MPI* = *H* * *A*
Indicator contribution-*C*	The contribution of an indicator to *MPI*, and its calculation formula is $C = \frac{{w_{i} CH_{i} }}{MPI}*100$, where, *CH*_*i*_ represents the deprivation ratio of indicator *i*, *w*_*i*_ represents the weight value of the index *i*
Indicator deprivation ratio-*X*	The ratio of the population with a deprived indicator to the total population

### Diversity analysis on multidimensional poverty’s characteristics

To some extent, China government attaches great importance to the balanced development of different rural regions, especially for those different types of historical poverty-stricken counties that other departments have compulsory duties to specially provide tilted support on assistance policies and resource.

Under the current conditions of Hechi City, each county is classified into different types according to its historic poverty characteristic, and *Theil*-*T* coefficient is introduced here to conduct inter-class and intra-class difference analysis, so as to measure the effectiveness and efficacy of different third-party departments on the anti-poverty development, also contributes them to taking specific measures to facilitate the next step. Compared with other difference analysis methods such as Gini coefficient and variable coefficient, *Theil*-*T* coefficient model can break down the overall differences of the research area into inter-regional differences and intra-regional differences (Hu et al. 2009), so that the gap or inequality between different types of counties can be better revealed. Employing the decomposability of *Theil*-*T* coefficient, this article measures the overall differences (*T*_*t*_), inter-regional differences (*T*_*r*_) and intra-regional (*T*_*a*_) differences under different poverty indicators respectively. As shown in formula (1)–(3).

1 $${\text{Overall}}\;{\text{difference:}}\,T_{\text{t}} = T_{\text{r}} + T_{\text{a}}$$

2 $${\text{Inter-regional}}\;{\text{difference:}}\,T_{\text{r}} = \sum\limits_{i = 1}^{n} {Y_{\text{i}} } \;{ \log }\frac{{Y_{i} }}{{P_{i} }}$$

3 $${\text{Intra-regional}}\;{\text{difference:}}\,T_{\text{a}} = \sum\limits_{i = 1}^{n} {Y_{i} } \sum\limits_{j = 1}^{n} {Y_{ij} \log \frac{{Y_{ij} }}{{p_{ij} }}}$$

where, *n* refers to the number of the classes after each county is been classified; *Y*_*i*_ represents the portion of the counties in Class *i* in the given indicator; *P*_*i*_ represents the multidimensional poverty headcount ratio of the given class *i*. *Y*_*ij*_ and *P*_*ij*_ represent the given indicator’s poverty contribution portion of county *j* in the class *i* of counties, and multidimensional poverty headcount ratio of the county *j* within class *i*, respectively. The larger the *Theil*-*T Index*, the bigger the differences of poverty characteristics, and vice versa.

## Results analysis on multidimensional poverty measurement of the research area

According to the above methodology of multidimensional poverty measurement and analysis, Hechi City’s poverty indexes, i.e., *H, A, MPI*, are achieved. And then, multidimensional poverty characteristic and their structural differences are revealed by use of spatial statistical analysis.

### The overall characteristic of multidimensional poverty of the research area

As stated in “Research area” section, *K* represents the number of multidimensional poverty cutoffs, which can be used to determine whether the household is under multidimensional poverty or not. In terms of different *K* values, the households’ poverty differences may be very big, as well as that of each indicator’s deprivation ratio. In this study, there are ten basic indicators, therefore, setting *K* = 1, 2,…, 10, we calculate the changes of *H*, *MPI* and *A* under given different *K* values of the research area, analyzing their changes, and then selecting a reasonable value of *K* to explore the poverty contributing factors according to the contribution of each indicator.

#### The characteristic of the study area’s multidimensional poverty indexes

As shown in Table 3, with the increase of *K, H* and *MPI* show a decreasing trend. When *K* = *8*, *H* and *MPI* being 0, indicating that there are no more than 8 basic indicators in the research area that are deprived extremely as poor counties. Under different *K* values, each identification indicator’s contribution degree changes little, showing that these indicators’ deprivation degrees are stable for the households. When *K*∈[1, 6], the contribution of ‘adults’ illiteracy’ is small; While *K* > 6, it increases, indicating that those households affected by this indicator are also affected by at least other five indicators at the same time. Since the ratio of the households under illiteracy poverty is small when *K* > 6, while the number is large when *K*∈[1, 6], so the contribution of the indicator is lower when assigning *K* a small value. Overall, the contribution of ‘fuel type’ is opposite to that of ‘adults’ illiteracy’, indicating that the number of households affected by the ‘fuel type’ is large.

**Table 3:** the whole study area’ poverty indexes and their contribution degrees under different *K* values

	Multidimensional poverty indexes			Basic identification indicator’s contribution degree									
*K*	*MPI*	*A*	*H*	School-aged children’s enrollment rate	Adults’ illiteracy	Member’s health	House safety	Drinking water’s safety	Drinking water’s availability	Sanitary facilities	Fuel type	Power access	Broadcasting access
1	0.29	0.26	0.99	0.03	0.09	0.29	0.25	0.05	0.05	0.07	0.14	0.01	0.03
2	0.29	0.34	0.85	0.03	0.08	0.3	0.26	0.05	0.05	0.08	0.12	0.01	0.03
3	0.25	0.39	0.64	0.03	0.08	0.28	0.28	0.06	0.05	0.08	0.11	0.01	0.03
4	0.19	0.45	0.42	0.03	0.08	0.26	0.3	0.06	0.06	0.08	0.09	0.01	0.03
5	0.13	0.52	0.25	0.03	0.08	0.25	0.32	0.06	0.05	0.07	0.08	0.02	0.04
6	0.07	0.6	0.12	0.03	0.08	0.24	0.33	0.06	0.05	0.07	0.07	0.03	0.05
7	0.03	0.7	0.04	0.04	0.1	0.27	0.31	0.06	0.04	0.06	0.06	0.03	0.05
8	0	0.81	0	0.05	0.13	0.27	0.29	0.05	0.03	0.05	0.05	0.03	0.05
9	0	0.82	0	0.06	0.12	0.25	0.25	0.04	0.04	0.04	0.04	0.04	0.04
10	0	0.18	0	0.02	0.02	0.05	0.05	0	0	0	0	0	0

Two basic indicators of ‘house safety’ and ‘members’ health’ are of great contribution degrees that has been maintained at around 30 % under different K values, showing when *K* is smaller, the proportion of households with the deprivation of housing and health indicators is larger; and vice versa, further indicating that households in high-dimensional poverty are of great deprivation in terms of these two indicators. In other words, when *K* is smaller, the covered poverty indicators are not so comprehensive; however, when *K* value is larger, the number of those households in higher dimensional poverty is too smaller to reflect the common poverty situation in the research area. Therefore, this article follows the UNDP standard, defining those households under about 30 % of deprived indicators as poverty-stricken ones (Lu 2012; Hu and Ou 2013), i.e., K = 10/3 ≈ 4. On the other hand, to scientifically select a cutoff, the ANOVA and logistic regression model, introduced by Gordon et al. (2000), Qi and Wu (2015), are also applied to find out which poverty cutoff could best distinguish the poor and non-poor. As a result of using F value and Chi^2^ value respectively, it can be seen that both the F value and Chi^2^ value are the biggest when *K* is defined as 4 for counting the valid deprivation indicators. Therefore, the following analysis are done when given *K* = 4.

#### Multidimensional poverty degree of the research area

**Fig. 3:**
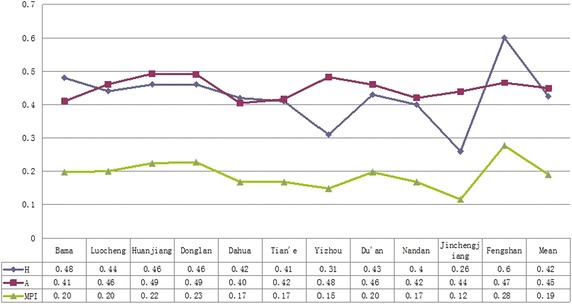
*H*, *A*, *MPI* of each county in the study area

According to the ‘Dimensional Aggregation’ algorithm in “Data sources and preprocessing” section, we obtain *MPI*, A and *H* of each county, respectively denoting the multidimensional poverty degree, poverty intensity and poverty occurrence. As shown in Fig. 3, the *MPI* of each county ranks from high to low: Fengshan > Donglan > Huanjiang > Luocheng > Bama > Du’an > Dahua > Nandan > Tian’e > Yizhou > Jincheng. As far as *H* is concerned, the three values, the average, the minimum and the maximum, are obviously different, showing that the proportion of multidimensional poverty varies greatly. *A* value of every county is about 0.40, showing that the difference among each county’s deprivation is smaller. In addition, it can be seen that high *H* is often accompanied by high *MPI*, indicating that proportion of the poor population with greater poverty is also higher.

**Fig. 4:**
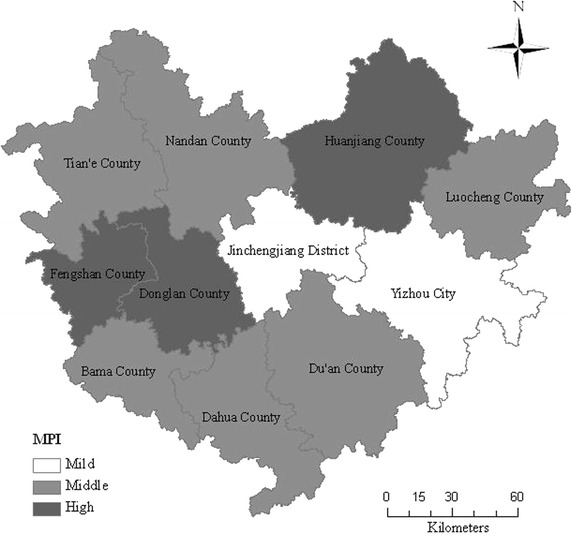
*MPI* spatial distribution for each counties when *K* = 4

According to each county’s *MPI* value, we use equal interval classification in ArcGIS (Atreshiwal 2012; Thongdara et al. 2012), dividing the 11 counties of the research area into three categories, as shown in Fig. 4. From an overall perspective, *MPI* shows a tendency of ‘higher in the rim and lower in the middle’. Regarding Jincheng County as the center, the surrounding counties are much poorer; instead of the central areas’ less poverty. Fengshan, Donglan and Huanjiang County belong to highly impoverished county, and Jincheng and Yizhou belong to mildly poor ones. *MPI* is increasing from south to north, also from west to east. In addition, those minority autonomous counties, i.e., Huanjiang, Du’an, Luocheng, Dahua and Bama, all belong to middle or high poverty.

#### Multidimensional poverty contributing factors

**Fig. 5:**
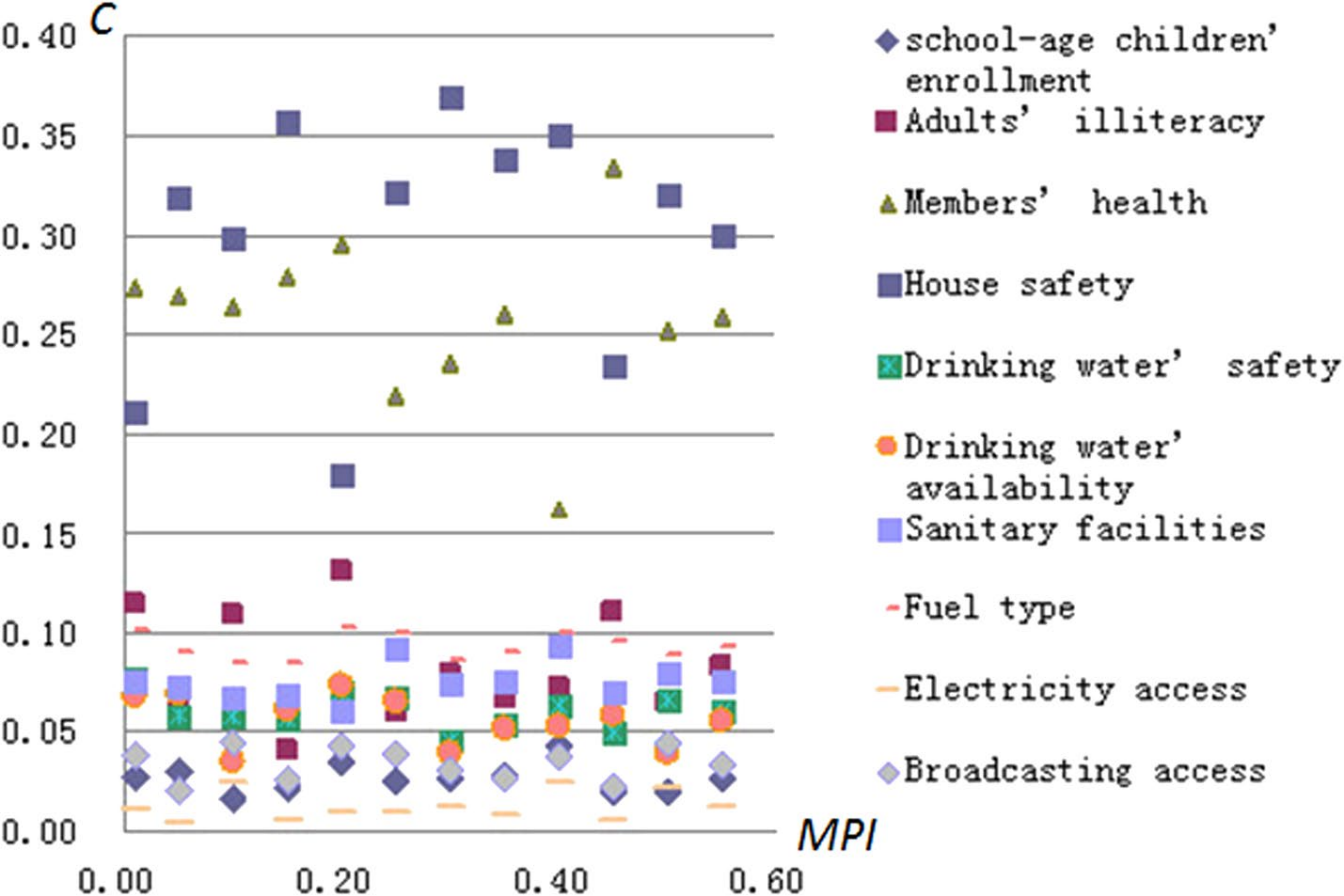
Contribution degree (*C*) of each indicator

Each county’s poverty factors may be different due to the *MPI* differences. The contribution degree, i.e., *C*, indicating each factor’s contributing to the county’s comprehensive poverty, is figured out by using ‘dimensional decomposing’ algorithm in “Data sources and preprocessing” section. Then, according to the average *C* value of each indicator, these indicators are divided into three classes in a descending order, namely, main poverty factors, general ones and minor ones, seeing Table 4, and Fig. 5 is the distribution of each indicator’s contribution degree.

**Table 4:** Poverty contributing factors

Indicator	Main poverty factors			General factors				Secondary factors		
	Unsafe housing	Poor health	Adults’ illiteracy rate	Dirty fuel type	School-age children enrollment rate	Sanitary facilities	Unsafe drinking water	Drinking water’s unavailability	Broadcasting access	Electricity access
Average value	0.408	0.360	0.059	0.021	0.019	0.018	0.014	0.013	0.008	0.003

As shown in Table 4 and Fig. 5, the three indicators, including ‘dangerous housing’ with contribution degree *C*∈[0.3, 0.4], ‘poor health’ with *C*∈[0.2, 0.3], and ‘adults’ illiteracy’ with *C*∈[0.05, 0.15], are classified as the main poverty factors due to their absolutely high contribution level. Similarly, ‘broadcasting access’ and ‘electricity access’ can be classified as minor poverty factors due to their *C* values between 0 and 0.05. The rest five indicators are classified as general poverty-contribution factors with their *C* values of 0.05–0.1.

From the perspective of spatial clustering, we also found that, *H* and *MPI* show a significantly high value aggregation in Fengshan, Donglan and Bama of the west, while there exists a significantly low value aggregation of *A* and *MPI* in the middle and southeast sections; what’s more, *A* shows a high spatial aggregation in Nandan, Luocheng and Dahua. All these mean that the middle and southeast of Hechi city are in medium poverty and the poverty differences among counties are small; on the contrary, the west and the north, Fengshan and Nandan, are in relatively deep poverty, as well as the middle and the southeast are of less poverty-stricken. The indicator of ‘children enrollment rate’ shows a very high value aggregation in Jincheng and Donglan of the middle, as well as a low value aggregation in the marginal area; ‘adults’ illiteracy’ shows a high value aggregation in Dahua of the south, and a low value aggregation in other counties. One of main poverty factors, ‘housing’, shows a high value aggregation in Yizhou of the east and Dahua of the south, rather than a significantly low value aggregation in Jincheng and Donglan of the middle. On the other hand, the ‘health’ indicator shows an insignificantly high value aggregation in Tian’e and Nandan of the northwest, while in other areas, it shows an insignificantly low value aggregation. In a word, in terms of poverty degree, the west of the study area shows a significantly aggregation, while the middle-east indicates a significant spatial difference; in terms of the poverty factors, Dahua and Bama of the south, and Nandan of the north showing significantly high value aggregation.

### Multidimensional poverty characteristics under different classes of social economic conditions

At present, Chinese government is conducting classified poverty-alleviation policies based on the poverty characteristics of each county and its locational conditions, and the trinity of poverty reduction policy that aids the poor in social development, industrial development, special aid-the-poor projects, respectively, has become an important measure to stimulate the development-oriented poverty reduction program for rural China. How to improve the local government behaviors to enhance the efficiency of poverty alleviation is one of the core problems for the poverty alleviation and development from now on. Therefore, when it comes to applying the *Theil* index’s great subgrouping decomposition strength to monitor the execution efficacies of different government departments’ efforts in aiding work, it will be very easy to find their vulnerability on aiding system, further to help take targeted measures in poverty alleviation.

**Fig. 6:**
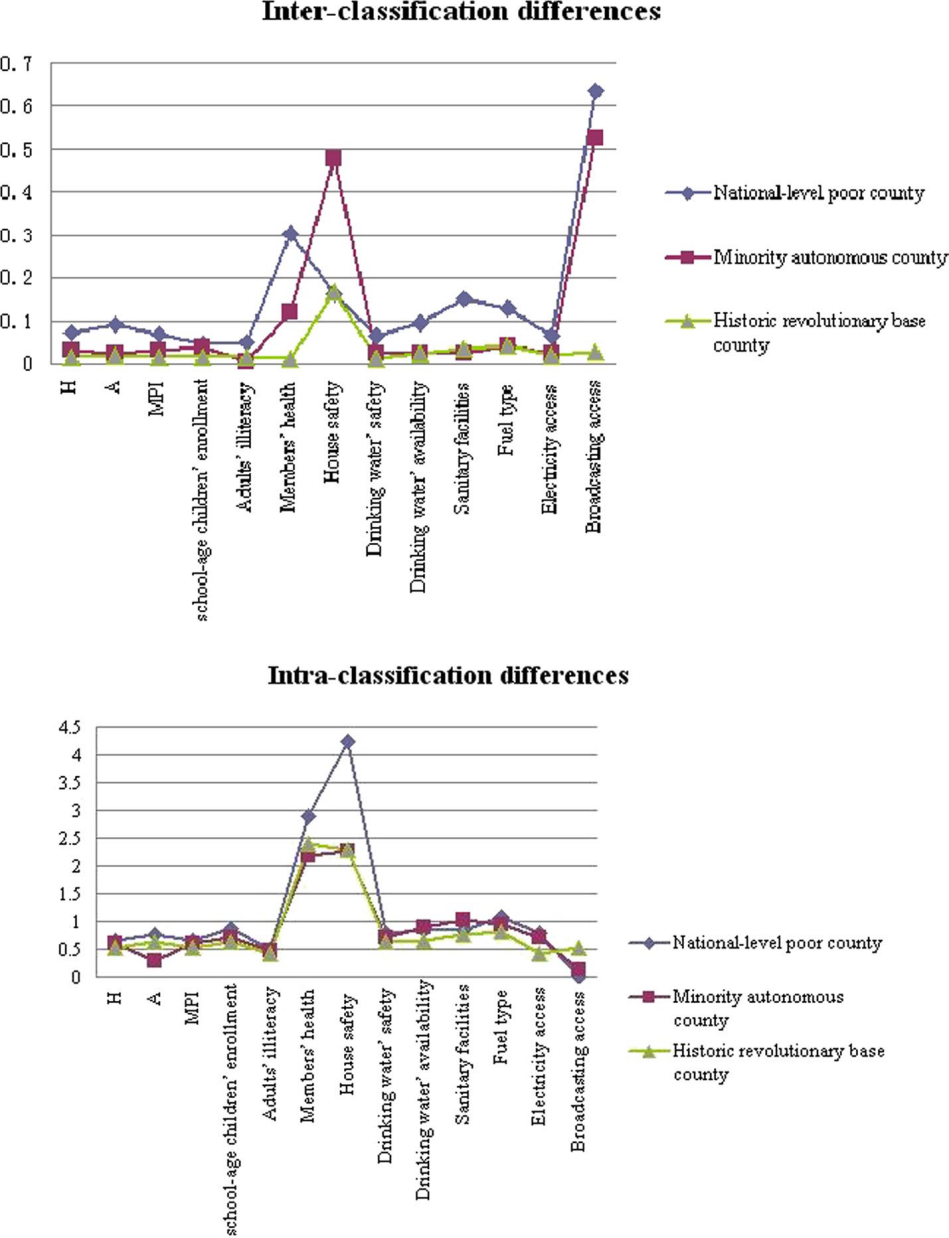
Intra-classification and inter-classification differences

In this context, we divide these counties of the study area into three classifications, according to each county’s locational conditions mentioned in “Related work” section, namely, national-level poverty-stricken county, minority autonomous county, historic revolutionary base county, adopting *T*_*t*_, *T*_*r*_ and *T*_*a*_*Theil* indexes in formula (1)–(3) to reflect the poverty diversity among intra- and inter-classifications, respectively. As shown in Table 5 and Fig. 6 that sheds a light on visually understanding the development differences, *T*_*rp*_ and *T*_*ap*_ represents *T*_*r*_ and *T*_*a*_ in percent terms, respectively.

**Table 5:** *Theil* indexes of three classifications

Classifications Indicators	National-level poverty-stricken county					Minority autonomous poverty-stricken county					Historic revolutionary-base poverty-stricken				
	*T*_*t*_	*T*_*a*_	*T*_*ap*_	*T*_*r*_	*T*_*rp*_	*T*_*t*_	*T*_*a*_	*T*_*ap*_	*T*_*r*_	*T*_*rp*_	*T*_*t*_	*T*_*a*_	*T*_*ap*_	*T*_*r*_	*T*_*rp*_
*H*	0.726	0.071	9.78	0.655	90.22	0.649	0.032	4.93	0.617	95.07	0.543	0.017	3.13	0.526	96.84
*A*	0.857	0.091	10.62	0.766	89.39	0.323	0.022	6.81	0.301	93.19	0.653	0.019	2.91	0.634	97.09
*MPI*	0.715	0.068	9.52	0.646	90.48	0.630	0.029	4.60	0.602	95.60	0.544	0.014	2.57	0.53	97.43
Children enrollment	0.922	0.046	4.99	0.876	95.01	0.76	0.038	5.00	0.722	95.00	0.641	0.015	2.34	0.626	97.66
Adults’ illiteracy	0.943	0.151	16.01	0.992	83.99	0.988	0.034	3.44	0.953	96.56	0.947	0.035	3.70	0.912	96.30
Family health	3.196	0.301	9.423	2.895	90.58	2.313	0.12	5.19	2.194	94.86	2.412	0.012	0.50	2.400	99.50
Housing	4.404	0.163	3.701	4.241	96.30	2.751	0.478	17.38	2.273	82.62	2.448	0.165	6.74	2.283	93.26
Drinking water’ safety	0.861	0.066	7.67	0.795	92.39	0.735	0.021	2.86	0.714	97.14	0.635	0.013	2.054	0.622	97.95
Drinking water’ availability	0.928	0.096	10.34	0.832	89.66	0.922	0.023	2.50	0.899	97.50	0.671	0.024	3.58	0.647	96.42
Sanitary facilities	1.004	0.152	15.144	0.852	84.86	1.038	0.023	2.22	1.015	97.78	0.794	0.035	4.41	0.759	95.59
Fuel type	1.198	0.130	10.85	1.068	89.16	0.991	0.042	4.24	0.949	95.76	0.856	0.041	4.79	0.815	95.21

In terms of the classification of national level poverty-stricken counties, the intra-classification differences of different indicators’ contributions are larger than those of other classes, except for the housing indicator; the inter-classification differences of different indicators are similar to those of other classes, except for the two indicators of drinking water’s safety and sanitary facilities. This indicates that the multidimensional poverty difference is quite larger among different poverty level of counties, while the internal difference among those counties at the same poverty level is comparatively smaller. In terms of the classification of minority autonomous counties, the intra-classification differences of indicators’ contributions are larger than those of historic revolutionary base classes, except for the indicators of adult’s illiteracy, drinking water’s safety and sanitary facilities. The inter-classification differences of different indicators are similar to those of other classes, except for drinking water’s safety and sanitary facilities. This indicates that the multidimensional poverty difference is greater between minority autonomous counties and non-minority autonomous counties, while the internal difference of the same classes is comparatively smaller. Overall, the poverty contributions of the two indicators, i.e., housing and adults’ illiteracy, show bigger intra-classification differences, while those of the other two indicators, drinking water’s availability and sanitary facilities, show bigger difference not only in intra-classification, but also in inter-classification. Which indicates there exist significant difference among those counties from three different classifications, as well as from the same classification, when regarding to the poverty degree of two indicators of drinking water’s availability and sanitary facilities.

Compared Table 3 with Table 5, it could be seen that, the contributing influence of different poverty identification indicators on the poverty degree is different from that on poverty diversity, also different under different classifications. As a whole, it seems that greater poverty contributing degree does not necessarily play a more important role in poverty diversity, due to the uneven development among the same classification of counties, as well as among different classification of ones. As a matter of fact, Table 3 comes from A–F measurement and component decomposition, resulting in three multidimensional poverty indexes and each indicator’s contribution degree to the three indexes, respectively; while Table 5 is from *Theil* index that reveals the diversities among different poverty indexes, as well as each indicator’s contribution degree to these diversities respectively, under different subjective poverty-stricken classifications. On the other hand, when given *K* = 4, the indicator ‘house safety’ becomes a greatest contributing factor to MPI. Correspondingly, it also shows a greatest *T*_*t*_ and a greatest *T*_*r*_ compared to other indicators under different classifications; However, *T*_*a*_ is an exception. Similarly, the second greatest contributing indicator, ‘member’s health’, and the least two greatest ones, ‘broadcasting access rate’ and ‘power access rate’, also show their corresponding ranks on *T*_*t*_ and *T*_*r*_, partly indicating that the poverty contributing factor has a positive correlation with *T*_*t*_, as well as with *T*_*r*_, the indicator w*ith a larger* poverty contributing degree having a larger *T*_*t*_ and *T*_*r*_.

### Multidimensional poverty characteristics under different topographic and geomorphic conditions

As a part of typical rocky desertification areas in Yunnan and Guangxi and Guizhou provinces of China, Hechi City’s natural environment constrains, e.g., topographic and geomorphic features, rocky desertification degree, etc., play an obvious role on the local social and economic development. In this context, the analysis on multidimensional poverty characteristics under different natural environments helps guide policies for effective poverty interventions, adapting to local conditions.

#### Multidimensional poverty characteristics under different topographic conditions

**Fig. 7:**
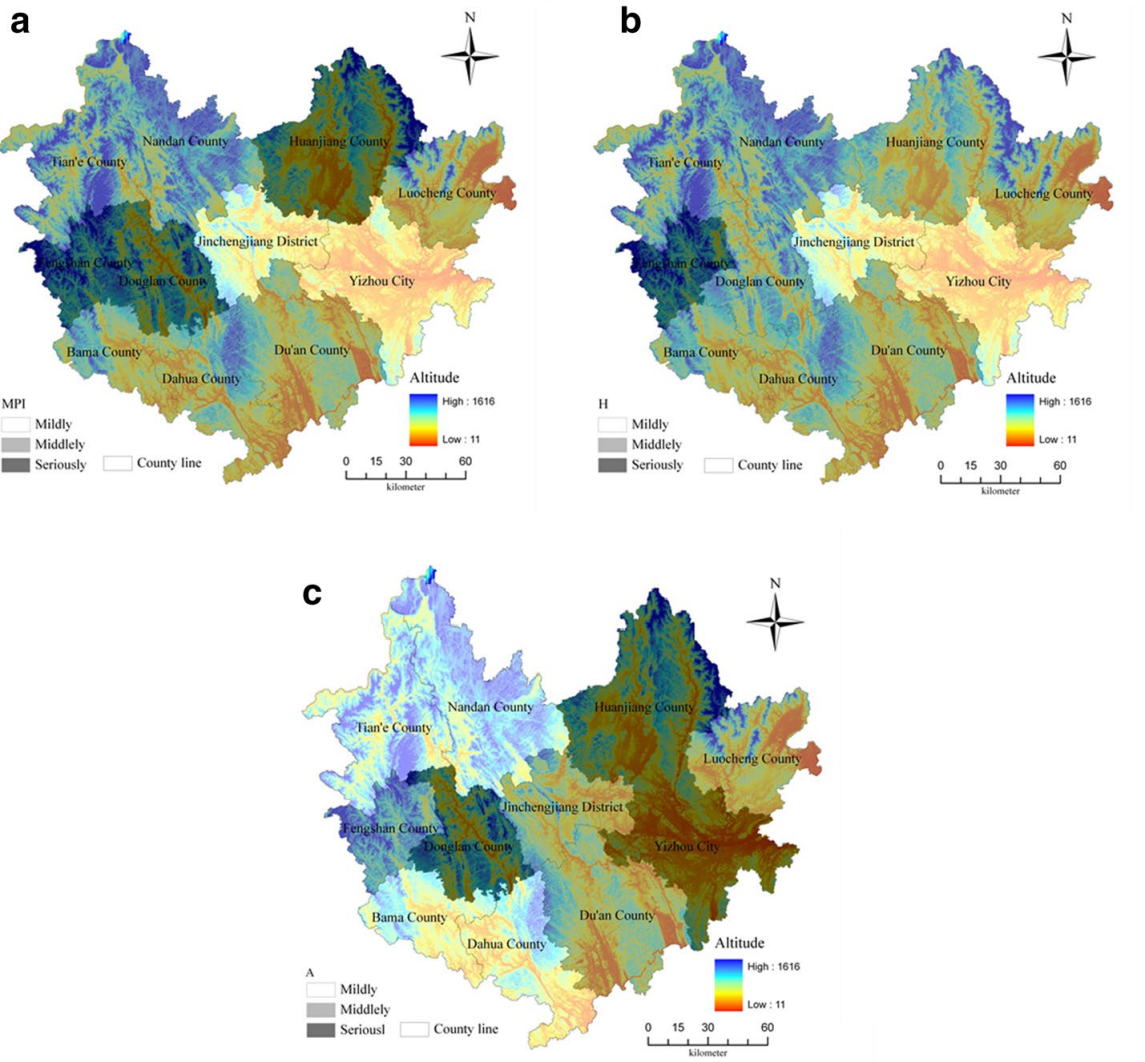
Overlay between DEM and *MPI*, *H*, *A*, respectively

Figure 7 shows the overlay distribution of each counties, between *MPI, H, A*, respectively, and the local elevation. From Fig. 7a, it can be seen that, mild poor counties, Jincheng and Yizhou, are of lower elevation and flatter terrain, compared with moderate poor counties, Nandan and Tian’e with a higher elevation; whereas, Fengshan and Donglan with relatively flat terrain are highly poor counties. So it could be partly said that comprehensive poverty degree has little relationship with elevation. From Fig. 7b, it can be seen that, Jincheng and Yizhou, with lower elevation and flatter terrain, have lower *H* values; whereas, Fengshan, with higher elevation and mountains terrain, has a larger *H* value. This indicating that, most of the households that are deprived of fewer poverty indicators, are mainly aggregated in relatively flat areas. Figure 7c shows that, all *A* values of Donglan, Huanjiang and Yizhou are the greatest, while these counties’ elevations are relatively lower; Jincheng, Du’an and Luocheng, all with middle *A*, are of lower elevation and flatter terrain; Nandan and Tian’e are located in the highest elevation and the most complex terrain, while they have the lowest *A*. All these show that *A* has a negative correlation with elevation, the higher the elevation, the lower the *A*.

On the other hand, topographic fragmentation degree of Hechi City is represented by the slope difference between the slope value of 90 m DEM and that of 1000 m DEM, and is classified by natural interval classification. By synchronously taking county as output unit to redraw it in ArcGIS and then spatially overlaying the fragmentation with *MPI, A* and *H*, respectively, it can be seen that, overall, the fragmentation degree has a positive correction with elevation, the more serious fragmentation comes with the higher elevation, and vice versa. As far as the relation of fragmentation degree with multidimensional poverty indexes is concerned, it can be similarly found that, *MPI*, *A* and *H* of each county, all are increasing with the increasing of fragmentation degree. The influence of fragmentation degree on these three indexes is larger in northwestern counties than that in central ones. All these are consistent with the spatial aggregation features of multidimensional poverty discussed in the above “The overall characteristic of multidimensional poverty of the research area” section.

#### Multidimensional poverty characteristics under different karst rocky desertification geomorphic conditions

**Fig. 8:**
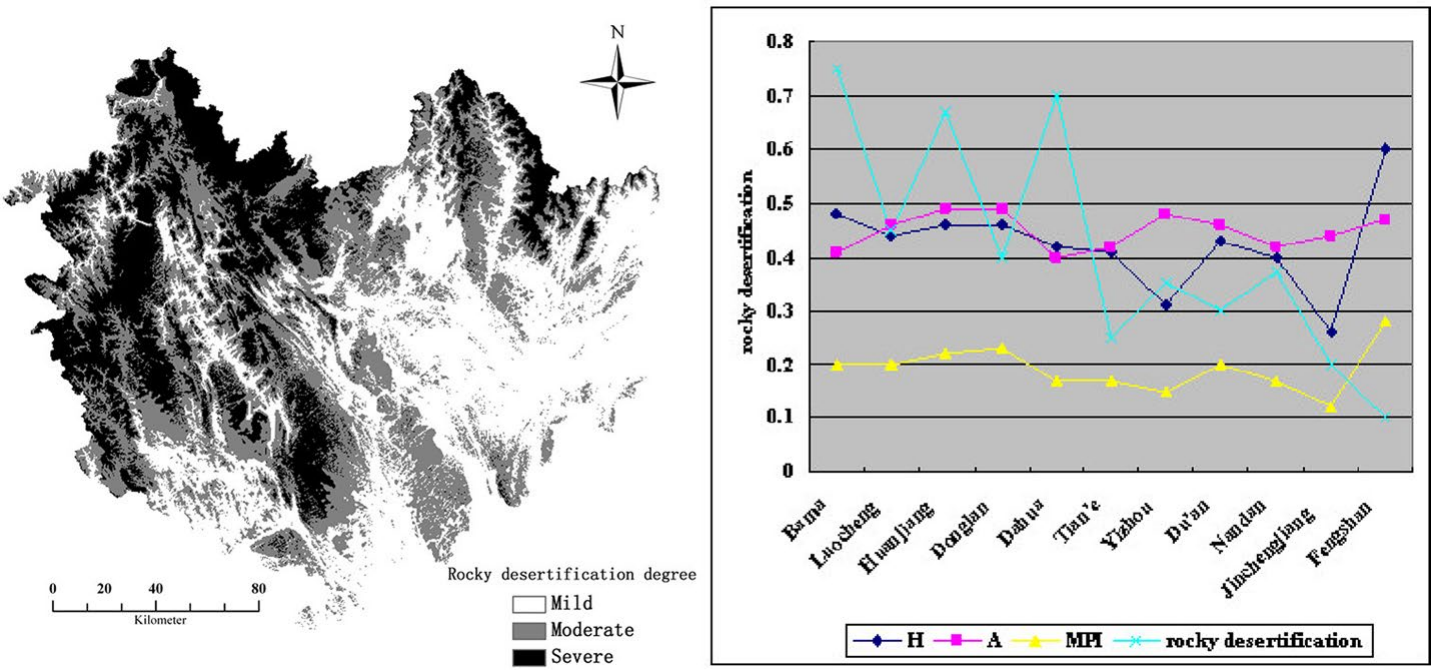
Karst rocky desertification Classification and its correlations with three poverty indexes, respectively

There exist serious contradictions among population, resources and environment in the Karst study area. We explore the relation between rocky desertification degree and multidimensional poverty of the study area. Similar to the above fragmentation degree’s representation, the rocky desertification degrees are also classified into three levels, i.e. mild, moderate and severe level, showed by using gray image and ArcGIS color rendering, respectively, as shown in Fig. 8.

Figure 8 reflects that rocky desertification is widely distributed in Hechi City and possesses typical regional differentiation characteristics. Six out of seven national-level poverty-stricken counties are located in moderate and severe level rocky desertification area. In term of *H*, those counties with higher rocky desertification, e.g., Bama, Dahua, Huanjiang, Yizhou etc., have higher *H* values, whereas counties with lower rocky desertification, like Tian’e, Fengshan, Jincheng etc., have lower *H* values. This partly indicates the area with high rocky desertification has high multidimensional poverty incidence, vice versa. Rocky desertification degree and multidimensional poverty incidence have positive correlation with each other.

In terms of *MPI* index, rocky desertification has the least influence on Jincheng, then Tian’e and Fengshan; however, its influence on MPI of the other counties is moderate level or above. This shows that most of the poverty-stricken households in the study area are subjected to rocky desertification. For index *A*, Huanjiang, Bama and Dahua have the largest *A* and the most severe rocky desertification, indicating that the households in those counties are deeply subjected to rocky desertification, and are deprived of more indexes than those in Tian’e, Fengshan and Jincheng, where there exist mild levels of rocky desertification.

Generally, *H*, *A* and *MPI* increase with the increasing of rocky desertification degree. This shows that the geographical distribution of poverty-stricken counties has an obviously positive correlation with rocky desertification. The reason is that there exists internal interaction effect between rocky desertification and poverty. The vulnerable ecological environment of the research area and human’s irrational activities cause current situation of karst rocky desertification, while human’s irrational activities are caused by poverty, vice versa. As a result, available resources become less and less, which further increases poverty. Therefore, poverty alleviation measures should be targeted, differentiated and precise, according to local natural environment and socioeconomic development conditions.

## Conclusion and discussion

Aiming at national ‘Precise Poverty Alleviation’ strategy of China, this article proposed a multidimensional poverty measure and analysis methodology, using GIS to measure poor households and their contributing factors, and taking 11 counties of Hechi City as study area, census archiving data of households as data source, respectively explored the poor’s multidimensional poverty characteristics under different geographic and socioeconomic conditions, as well as their spatial distribution diversities.

The main methodology includes: A set of multidimensional poverty measure indicators with Chinese Characteristics was proposed, consisting of 4 dimensions and 10 basic indicators; A multidimensional poverty measure model based on A–F double cutoffs was developed to evaluate the poor’s multidimensional poverty characteristics; A GIS spatial analysis method was introduced to descript the spatial diversity under different geographic and socioeconomic conditions.

The case test of 11 counties in Hechi City showed that, firstly, each county existed at least four respects of poverty, the whole poverty level showed the spatial trend of surrounding higher versus middle lower, as well as that of northern higher versus southern lower; Secondly, the main poverty contributing factors of the research area were followed in descending order: unsafe housing, family health and adults’ illiteracy. Thirdly, under three kinds of socioeconomic classification systems, the intra-classification differences of *H, A* and *MPI* are greater than their inter-classification differences. Fourthly, these three multidimensional poverty indexes increased with the increasing of rocky desertification degree and rocky desertification of the study area, *A* having an obvious negative relation with the study area’s elevation.

In closing, this article tried to analyze multidimensional poverty from the perspective of combining socioeconomics and human geography, developing multidimensional poverty measurement and analyzing methodology with Chinese Characteristics, realizing GIS application in multidimensional poverty identification and measurement. Such efforts not only provide scientific basis for precisely targeting poor people and aiding decision-making for further special poverty alleviation of China, but also offer references for both domestic and foreign related research.

It is noteworthy that, due to data limit, this article could not be able to monitor the multidimensional poverty change at a series of spatial and temporal scales, and conducting spatial analysis at county level is not so sufficient to support for the village- level ‘entire-village advancement’ poverty reduction work of China, all these are also our research directions to the next work.

Further, there still exists some issues on how to decide a ‘right’ deprivation cutoff, poverty line and weights, although there are many studies in line with {0,1} dichotomy of the derivation cutoff and equal weights in A–F applied studies, considering poverty also as a matter of degree rather than an attribute that is simply present or absent for individuals in the population, we had also tried to adopt the fuzzy measure to address the poverty-line robust, and the result is not so variable due to the relative little difference when compared to a simple {0,1} dichotomy. To be specified, three poverty indexes, *A*, *H*, *MPI*, have not obvious changes until *K* = 6, when it may not make much sense for the practical work due to the improper *K* value that is the ideal value of 4. So only for the purpose of simplification, this paper adopted {0, 1} dichotomy estimates as the derivation cutoff. We are conscious of the limitations that this test is only the preliminary work, and obviously, exploring cutoff robustness of the study area needs a further improvement in the future work.

**Fig. 9:**
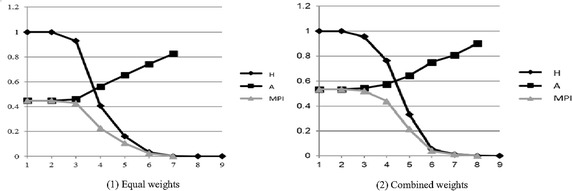
The correlations between K and different poverty indexes with two different weighting methods

On the other hand, to examine the possible result diversities that result from various weights, this article also adopted two different kinds of weighting methods, equal weights and combined weights that integrate subjective equal weight and objective entropy one based on game theory, to make a comparative analysis. Essentially, the combined weight model based on the game theory is devoted to finding a consensus or compromise among different weights, and the most reliable weights can be represented in a form of optimized weight set by minimizing the respective deviation between the possible actual weight and various basic weights. i.e., in this paper, based on game theory, the combined weight can be defined as an optimal weight between equal weight method and entropy value method. The specific calculation can refer to such literatures as Wang and Qian (2015). From the two different weight, we found that the results are various. For example, randomly taken out one case study, the two results are shown in Fig. 9. When given *K* = 1–3 and 6–9, the three indexes, *M, H, MPI, and their* differences calculated by different weights have no significant changes, respectively. When given *K* = 3–6, *H* and *MPI* have been greatly changed with different *K*; correspondingly, their differences under different weighting methods have also changed greatly. The most obvious is in the case of *K* = 4–5, when the differences of all the three indexes under different weighting methods began to mutate, especially *MPI* changing its value from 0.227 under the equal weight method to 0.438 under combined weight method, indicating that almost all the households in this case have been deprived from at least three indicators. From the beginning of *K* = 4, the number of the households under the multidimensional poverty becomes less. Similarly, when given *K* = 4, we also found the contribution degrees of different indicators had also changed under these two different weighting methods. To be specified, under the equal weights, the top three contributing factors include house safety, health, and fuel type; however, taken the combined weights into consideration, the ranking is changed into the order of house safety, health, and sanitary facilities. From the above, it can be known the results may be different due to different weighting methods and different poverty cutoffs.

**Fig. 10:**
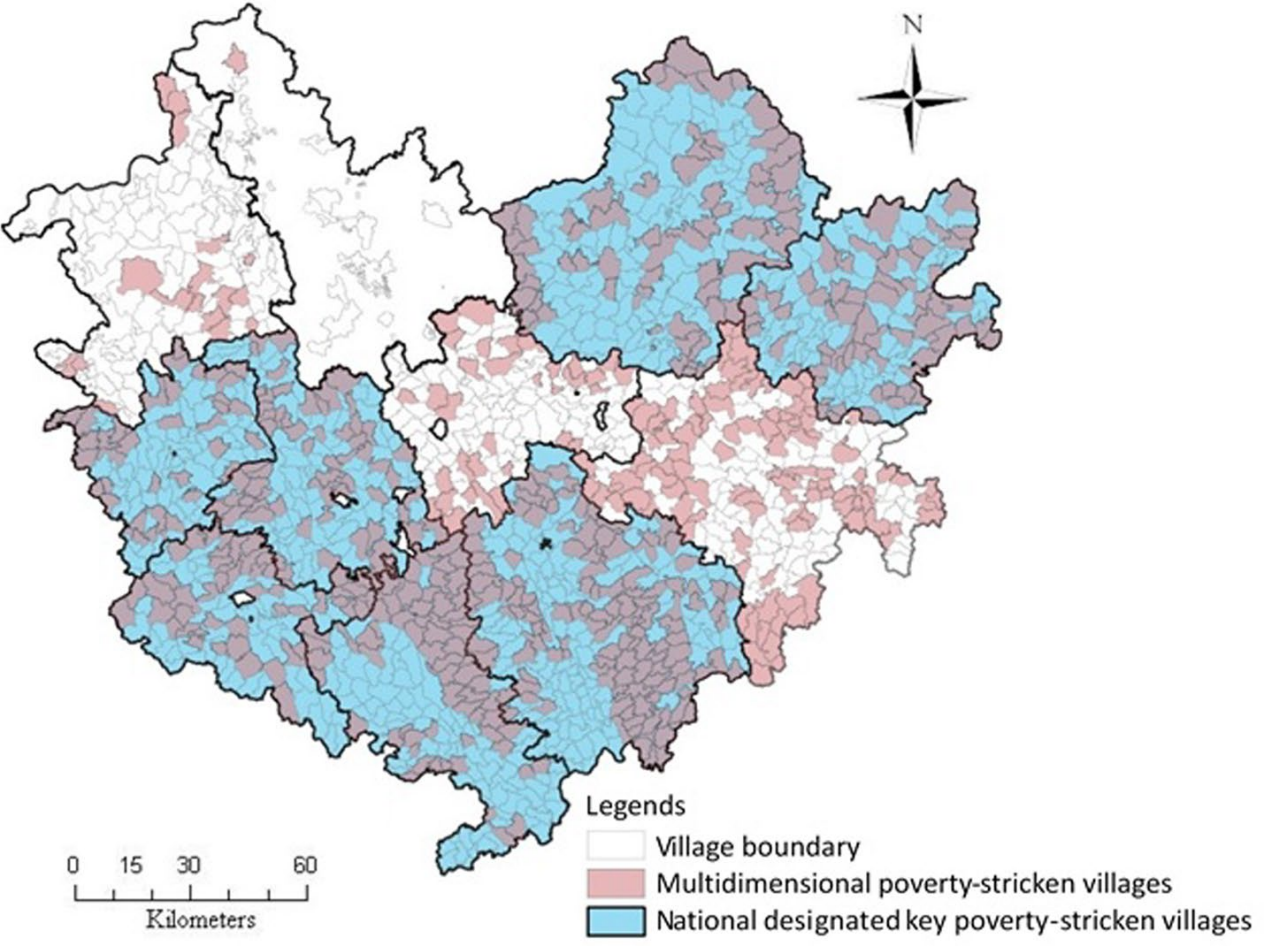
Overlap between identification results and national designated ones under equal weighting method

In terms of the identification accuracy, we also adopted ‘overlap ratio’ of the poverty-stricken villages between multidimensional profile and monetary profile as the test indicator aiming to the difference result only from different weighting methods. Here, we used equal interval classification in ArcGIS, dividing all the villages of the research area into three categories, similarly as shown in “Multidimensional poverty degree of the research area” section. We examined how the highly and moderately poverty-stricken villages match with the national assigned key villages list. As shown in Fig. 10, compared to the list of national designated key poverty-stricken villages that are recognized according to the 2300 Chinese RMB Yuan of income poverty line, there are 66.3 % overlap ratio, while this number changed to 64.6 % in the condition of combined weighting method. In this case, it seems that the two approaches produced similar results, and the difference is not significant with regard to multi-poverty.

However, these test results from the above do not mean that it is tenable in other cases. After all, this is the primary test only in one case, as well as only the comparison between two methods. Therefore, adopting different approaches from the statistical perspectives of correlation, concordance and pairwise comparisons to systematically check various weighting methods need much improvement in depth.
